# Construction of an inert framework in porous SiO_*x*_/Si anodes for high-performance Li-ion batteries

**DOI:** 10.1039/d6ra03303b

**Published:** 2026-06-02

**Authors:** Yu Cao, Qingxu Zhang, Jiucong Liu, Xizheng Liu

**Affiliations:** a Tianjin Key Laboratory of Advanced Functional Porous Materials, Institute for New Energy Materials & Low-Carbon Technologies, School of Materials Science and Engineering, Tianjin University of Technology Tianjin 300384 P. R. China xzliu@tjut.edu.cn; b Key Laboratory of Flexible Optoelectronic Materials and Devices (Ministry of Education), School of Optoelectrical Materials and Technology, Jianghan University Wuhan 430056 China xzliu@jhun.edu.cn

## Abstract

Si-based anodes with porous structures demonstrate improved electrochemical performance in Li-ion batteries; however, the collapse of pores during cycling remains a major challenge for practical applications. Herein, we report the construction of a hierarchical porous SiO_*x*_/Si composite anode with an inert Mg_2_SiO_4_ skeleton to stabilize the porous structure during reversible Li-storage. The Li-active components SiO_*x*_/Si are dispersed within a continuous rigid framework, enabling more effective confinement of volume changes and better preservation of structural integrity. The successful synthesis of porous SiO_*x*_/Si with an inert Mg_2_SiO_4_ framework is confirmed by XRD and XPS, indicating uniform distribution of active components within the framework. As a result, the specific capacity is improved from 519 mAh g^−1^ to 775 mAh g^−1^ after 100 cycles. The lithium-ion diffusion coefficient (*D*_Li^+^_) reaches 3.42 × 10^−10^ cm^2^ s^−1^, indicating that the stabilized hierarchical porous structure promotes efficient Li^+^ transport throughout repeated charge–discharge processes. The inert Mg_2_SiO_4_ framework remains stable even after long-term cycling. This study offers a new material design strategy for Si-based anodes, which will promote the practical application of porous structured Si anodes.

## Abstract 2

A new porous SiO_*x*_ materials with inert framework is proposed, the structure stability of porous during repeated lithiation/delithiation cycles has been distinctly improved and thus an enhanced cycle performance.

## Graphical abstract

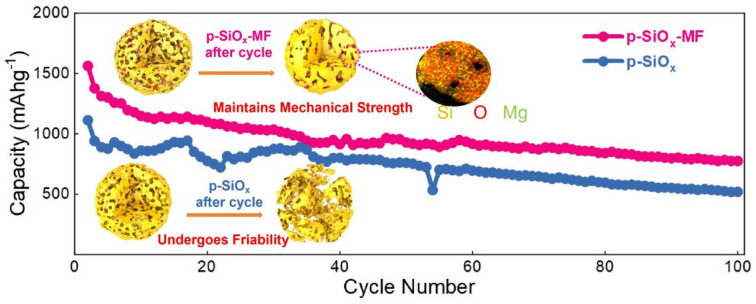

## 1 Introduction

Current lithium-ion batteries (LIBs) technology has witnessed great success in both academia and industry, and its rapid advancement has strongly driven progress in related fields such as portable electronics, new energy vehicles, smart healthcare, and artificial intelligence.^1–4^ Extending into new emerging applications is driving the development of LIBs toward higher energy density, improved cycling stability and enhanced safety performance.^5–7^ The present limitation for energy density is largely constrained by the commercial graphite anode with a low theoretical capacity (372 mAh g^−1^).^8–10^ To overcome this bottleneck, high-capacity Si-based materials have been extensively investigated as next-generation anodes owing to their high theoretical capacity and improved cycling stability. The significant volume expansion and pulverization of Si-based anodes have been commonly mitigated by design and fabrication of porous structured materials.^11–15^

Porous Si anodes have been developed through various methods. Chemical etching produces high-surface-area nanoporous Si with improved cycle stability.^16,17^ Hydrothermal synthesis creates mesoporous spheres that relieve volume expansion. Template methods yield 3D interconnected structures with superior rate capability. Electrochemical corrosion realizes graded porous structures with enhanced ion transport, while magnesiothermic reduction converts biomass silica into low-cost composites.^18,19^ In previous work, we have developed hierarchical porous SiO_*x*_ (p-SiO_*x*_) anodes derived from natural diatomite, which feature a unique architecture with crystalline Si domains embedded in an amorphous SiO_2_ matrix and an interconnected macro/mesopores structure. Significantly improved performance is achieved compared with conventional SiO_*x*_.^20^ Nevertheless, long-term cycling stability remains unsatisfactory due to the gradual degradation of the porous structure during prolonged cycling.^21,22^ During continuous lithiation and delithiation processes, porous Si-based materials undergo dramatic volume fluctuations, which induce severe internal mechanical stress and cause bending, cracking, and fracture of thin pore walls. Repeated volume change also leads to gradual pulverization and agglomeration of silicon particles, which fill the original pore voids. Meanwhile, the unstable SEI layer repeatedly ruptures and regenerates upon cycling, further blocking pore channels.^23–25^ All these factors jointly trigger irreversible pore collapse and structural densification, resulting in fast capacity fading and poor cycling performance of conventional porous Si anodes.^26–28^

Recent studies show that constructing robust internal scaffolds within Si-based anodes can effectively enhance structural integrity even under deep discharge–charge cycles. Zhong *et al.* reported a two-step magnesiothermic reduction to produce carbon-coated MgSiO_3_-doped SiO_*x*_ (MgSiO_3_-SiO_*x*_@C), which exhibits 62.7% capacity retention after 150 cycles.^29^ Using a two-step CVD method, Yang *et al.* fabricated a Si/C composite supported by porous carbon, which overcomes mechano-kinetic limitations and achieves 80% capacity retention over 1700 cycles.^30^ Broken Si particles can be held by the carbon support. All these advances greatly improve the stability and reversibility of Si-based composite anodes.^31–33^ However, complicated processes or toxic agents during material preparation limit their large-scale application. It is still highly desirable to develop a facile and scalable method that effectively stabilizes the porous structure during repeated cycling.^34–36^

Herein, we successfully construct a hierarchical porous SiO_*x*_/Si composite anode with an inert Mg_2_SiO_4_ rigid framework through a simple magnesiothermic reduction route using natural diatomite as precursor. The introduced inert skeleton effectively restrains volume expansion, prevents pore collapse and maintains structural integrity during long-term cycling. Benefiting from the stable porous architecture, the optimized porous SiO_*x*_ within a mineral framework (p-SiO_*x*_-MF) electrode delivers a high reversible capacity of 775 mAh g^−1^ after 100 cycles, much higher than the 519 mAh g^−1^ of the pristine porous SiO_*x*_. Meanwhile, the lithium-ion diffusion coefficient reaches 3.42 × 10^−10^ cm^2^ s^−1^, demonstrating fast ion transport kinetics. This work offers a low-cost, facile and scalable strategy to stabilize porous Si-based anodes, which is promising for promoting their practical application in high-performance lithium-ion batteries.

## 2 Experimental sections

### 2.1 Synthesis of hierarchical porous structured SiO_*x*_ anode

All chemicals were commercial and used without further purification. The magnesium powder, diatomite, and sodium chloride were mixed in a molar ratio of 1 : 1.5 : 5 and subjected to ball milling for 30 min to achieve homogeneity. Subsequently, the blended powder was transferred into a crucible. A layer of NaCl was first spread at the bottom of the crucible, followed by the uniform placement of the mixed powder, and then covered with another layer of NaCl. Thermally treated in a tube furnace under argon atmosphere with a heating ramp of 5 °C min^−1^ to 850 °C, where it was held for 6 h before cooling to room temperature. The resulting product was etched in 0.5 mol L^−1^ HCl for 12 h to obtain the porous p-SiO_*x*_-MF.

### 2.2 Material characterization

The structure of materials was studied *via* X-ray diffraction (XRD, Rigaku, Miniflex 600) by using Cu Kα (*λ* = 0.15418 nm). The morphology and structure were observed by scanning electron microscopy (SEM, Verios 460L, FEI) and transmission electron microscopy (TEM, Talos F200X, FEI). Valence states of each element were carried out using X-ray photoelectron spectroscopy (XPS, Escalab 250Xi, Thermo Fisher Scientific). The specific surface area and pore size distributions were taken by measuring N_2_ adsorption desorption isotherms at 77 K from a Quadrasorb SI analyzer and the calculation using the BET method.

### 2.3 Electrochemical measurements

The electrode materials were assembled into CR2032 coin cells for studying the electrochemical performance. A homogeneous slurry was prepared by mixing 70% active material, 15% KB, and 15% CMC binder and coated on Cu foil current collector. The mass loading was controlled to be about 1.2 mg cm^−2^. Celgard 2400 was as separator and 1.0 M LiPF_6_ in EC : DMC = 1 : 1 vol% with 5.0% FEC was used as electrolyte. The cells were assembled in an argon-filled glovebox. Cyclic voltammetry (CV) was performed on a CHI 760E electrochemical workstation with a scan rate of 0.5 mV s^−1^. At constant temperature of 30 °C. Galvanostatic intermittent titration technique (GITT) measurements were carried out on half cells within 0.05–2 V (*versus* Li^+^/Li). A titration current of 200 mA g^−1^ was used for 30 min along with the relaxation time of 3 h to reach the quasi-equilibrium potential.

## 3 Results and discussion

### 3.1 Synthesis and structural evolution

The synthesis process begins with diatomite as the precursor, followed by a magnesiothermic reduction reaction using metallic magnesium powder to convert silica into SiO_*x*_. This exothermic reaction causes a rapid temperature increase, leading to the quick volatilization of the reducing agent, magnesium. Sodium chloride (NaCl) plays a crucial role in this process: its melting point of 801 °C facilitates the transformation from a solid–solid reaction to a solid–liquid reaction at the reaction temperature of 850 °C, thereby promoting thorough reactions among different reactants. Additionally, a double-layer symmetric molten salt structure is employed to eliminate temperature gradients within the reaction, converting the non-uniform heat distribution caused by unidirectional heat transfer into a homogenized thermal field throughout the entire system. This approach prevents explosive escape of magnesium vapor due to local overheating and significantly enhances the crystallinity and uniform distribution of Mg_2_SiO_4_ in the final product. After the reaction, precise control of hydrochloric acid concentration and stirring time during post-processing steps allows for the regulation of the magnesium silicate content. Controlling the magnesium silicate content ensures the stable retention of macropores and the residual presence of micropores.

**Fig. 1:**
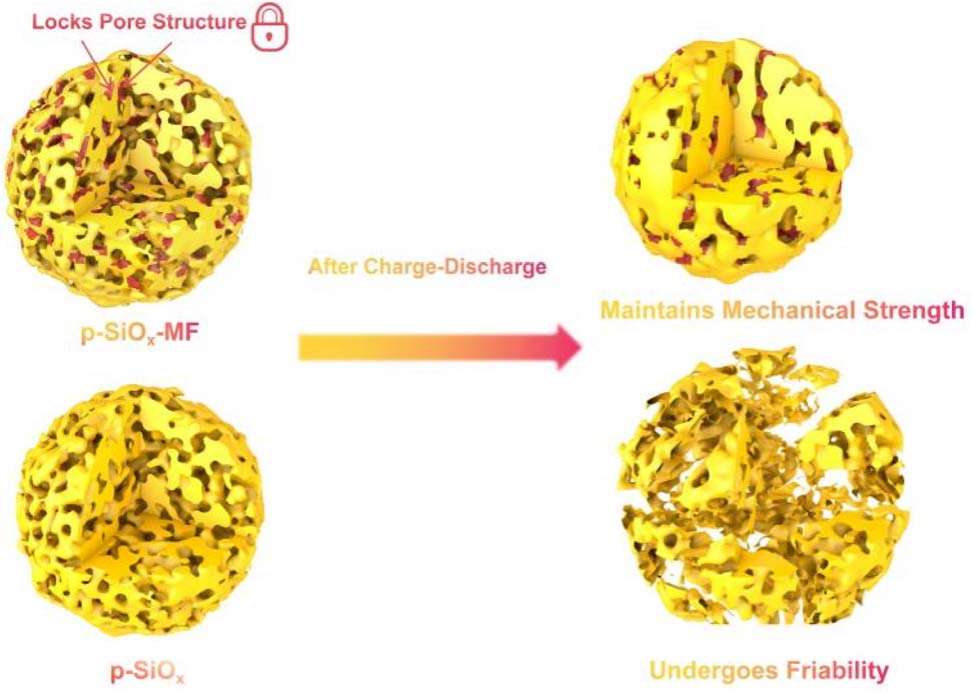
Schematic illustration the structure evolution of porous SiO_*x*_ with inert framework and normal porous materials after encountering with discharge/charge cycles.

As shown in Fig. 1, the structural stability and mechanical properties of p-SiO_*x*_-MF with an inert framework (Mg_2_SiO_4_) and p-SiO_*x*_ without an inert framework are compared before and after charge–discharge cycles. p-SiO_*x*_-MF exhibits a stable porous structure, with its pores “locked” by the inert framework, ensuring structural integrity during the charge–discharge process. In contrast, while p-SiO_*x*_ also possesses a porous structure, it lacks such stability. The situation after charge–discharge cycles is depicted: p-SiO_*x*_-MF maintains its mechanical strength and overall structural integrity even after undergoing these cycles, whereas p-SiO_*x*_ experiences friability, resulting in a fragmented and discontinuous structure that indicates a significant decrease in mechanical strength. This demonstrates that p-SiO_*x*_-MF exhibits superior structural stability and mechanical performance during charge–discharge processes, avoiding structural damage caused by friability, thanks to its “locked” pore structure mechanism.

### 3.2 Structural and compositional characterization

**Fig. 2:**
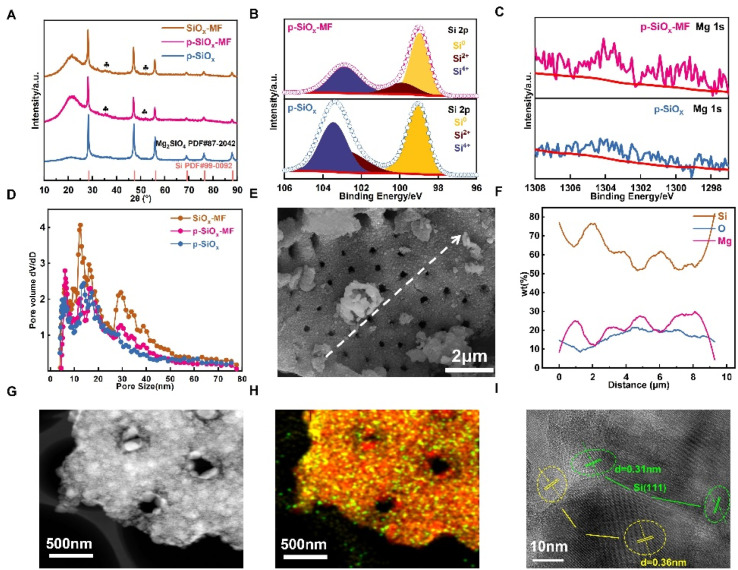
Characterizations of the as-prepared porous SiO_*x*_. (A) XRD patterns, (B) XPS of Si 2p and (C) Mg 1s; (D) pore size distribution. (E) SEM image and (F) linear sweep of elemental distribution of p-SiO_*x*_-MF. (G) TEM image, (H) corresponding EDS mapping, and (I) HR-TEM of p-SiO_*x*_-MF.

Fig. 2A shows the XRD patterns of SiO_*x*_-MF, p-SiO_*x*_-MF, and SiO_*x*_. The diffraction peaks at approximately 20°, 30°, and 45° are consistent with the standard PDF card for Mg_2_SiO_4_ (PDF #87-2042), indicating the presence of magnesium silicate in the samples. Additionally, the peak at around 26° corresponds to the PDF card for silicon (PDF #99-0092),^37^ confirming the existence of crystalline silicon in the material. These results suggest that the SiO_*x*_ materials contain both magnesium silicate and silicon phases, which could be attributed to the synthesis conditions such as temperature and precursor ratios. The XPS spectra of Si 2p reveal three distinct peaks corresponding to Si^4+^, Si^2+^, and Si^0^ states, which are indicative of the different oxidation states of silicon in the samples (Fig. 2B and S2). This suggests that the SiO_*x*_ materials have a mixed valence state of silicon, likely due to the formation of various silicon oxides during the synthesis process. The Mg 1s spectrum shows a single peak at approximately 1303 eV, confirming the presence of magnesium in the samples (Fig. 2C). The pore size distribution curve obtained from BET analysis indicates that the SiO_*x*_-MF and p-SiO_*x*_-MF samples exhibit a higher pore volume compared to the p-SiO_*x*_ sample, with pore sizes ranging from 2 nm to 80 nm (Fig. 2D). This enhanced porosity is beneficial for improving the electrochemical performance of the materials by providing more active sites for Li^+^ insertion/extraction and facilitating electrolyte diffusion.

The morphology and elemental distribution of the p-SiO_*x*_-MF sample were further investigated using SEM and TEM with energy-dispersive X-ray spectroscopy (EDS). The SEM image shows the porous structure of the p-SiO_*x*_-MF sample, with an average pore size of approximately 2 µm (Fig. 2E). The linear sweep of elemental composition along the dashed line confirms the uniform distribution of Si, O, and Mg throughout the sample, which is crucial for ensuring consistent electrochemical behavior across the material (Fig. 2F). The SEM images and elemental composition line scans in Fig. S3 demonstrate the impact of varying magnesium silicate content on the pore structure. As the magnesium silicate content decreases, its supporting and stabilizing role for the pores diminishes, leading to instability and deformation of the pore structure, thereby disrupting the uniformity of the pore distribution. Furthermore, the loss of the supportive magnesium silicate framework results in a decline in mechanical properties such as compressive strength and toughness.

Fig. S2A–C illustrate that with the reduction in magnesium silicate, the surface becomes increasingly rough and uneven. The line scan data confirm a significant decrease in magnesium content, particularly in p-SiO_*x*_, where magnesium is almost undetectable. The TEM image (Fig. 2G) provides further insight into the microstructure of the p-SiO_*x*_-MF sample, revealing a well-defined porous network that can accommodate volume changes during cycling. The EDS mapping (Fig. 2H and S4) reveals the spatial distribution of Si, O, and Mg elements within the sample, confirming the presence of these elements in the material. The HR-TEM image (Fig. 2I) shows the lattice fringes of the Mg_2_SiO_4_(210) plane with a *d*-spacing of 0.36 nm, providing direct evidence of the crystalline structure of the magnesium silicate phase in the sample. This crystalline structure contributes to the mechanical stability of the material, preventing structural degradation during repeated charge–discharge cycles. The comprehensive characterization of the as-prepared SiO_*x*_ materials confirms the successful synthesis of a porous SiO_*x*_ material with a mixed valence state of silicon and the presence of magnesium silicate, making it promising for applications in lithium-ion batteries and other electrochemical devices.

### 3.3 Electrochemical performance

**Fig. 3:**
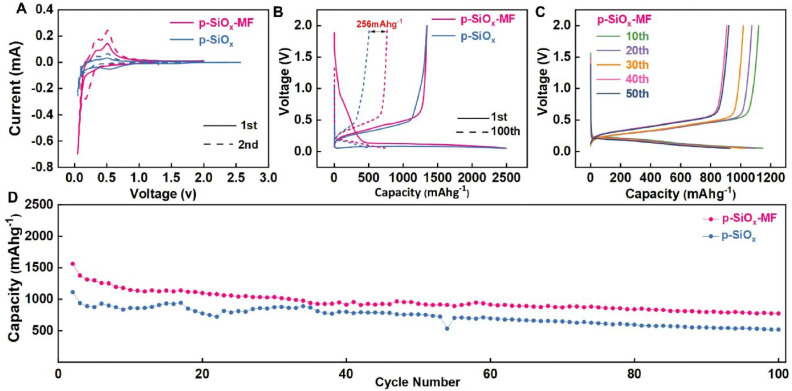
Electrochemical performance. (A) CV curves of p-SiO_*x*_-MF and p-SiO_*x*_. (B) Voltage profiles of the 1st and 100th charge/discharge cycles. (C) Selected cycles of charge/discharge curves of the p-SiO_*x*_-MF at 0.2 A g^−1^. (D) Cycle performance of SiO_*x*_ at 0.2 A g^−1^.

Fig. 3A displays the cyclic voltammetry (CV) curves of p-SiO_*x*_-MF and p-SiO_*x*_ under different scan cycles. Both materials exhibit similar redox peaks during the first scan (solid line) and second scan (dashed line), indicating comparable electrochemical activity. However, the CV curve of p-SiO_*x*_-MF shows a more stable current response in the second scan, suggesting better cycling stability and lower polarization effects. This can be attributed to the porous structure of p-SiO_*x*_-MF, which enhances the electrochemical activity and stability of the electrode material.

Fig. 3B and C collectively provide insights into the charge/discharge curves of p-SiO_*x*_-MF and p-SiO_*x*_. Fig. 3B compares the voltage–capacity profiles during the 1st and 100th charge/discharge cycles. During the initial cycle, the discharge capacities of p-SiO_*x*_-MF and p-SiO_*x*_ are comparable. At 100th cycle, p-SiO_*x*_-MF demonstrates a higher discharge capacity (approximately 256 mAh g^−1^), indicating superior cycling stability and capacity retention capability. Fig. 3C illustrates the representative charge/discharge curves of p-SiO_*x*_-MF at a current density of 0.2 A g^−1^ for the 10th, 20th, 30th, 40th, and 50th cycles. The charge/discharge curves of p-SiO_*x*_-MF show high repeatability and stability across these cycles, further confirming its excellent electrochemical performance and structural stability over multiple cycles. Additionally, the shape and position of the curves change minimally with increasing cycle numbers, which is consistent with the findings from Fig. 3B. This consistency underscores the superior cycling stability of p-SiO_*x*_-MF, reinforcing the notion that its porous structure contributes to enhanced electrochemical activity and stability.

Fig. 3D displays the cycling performance of p-SiO_*x*_-MF and p-SiO_*x*_ at a current density of 0.2 Ag^−1^. During the initial cycles, the capacity of p-SiO_*x*_-MF drops rapidly but then stabilizes, maintaining around 775 mAh g^−1^ after 100 cycles. In contrast, the capacity of p-SiO_*x*_ continues to decline throughout the cycles, eventually reaching approximately 500 mAh g^−1^ after 100 cycles. This indicates that p-SiO_*x*_-MF possesses better cycling stability and capacity retention, It due to its porous structure and enhanced structural stability. In summary, p-SiO_*x*_-MF exhibits significant advantages in electrochemical performance, including higher initial capacity, better cycling stability, and improved capacity retention (Table S1). These results suggest that p-SiO_*x*_-MF is a promising electrode material for high-performance energy storage applications.

**Fig. 4:**
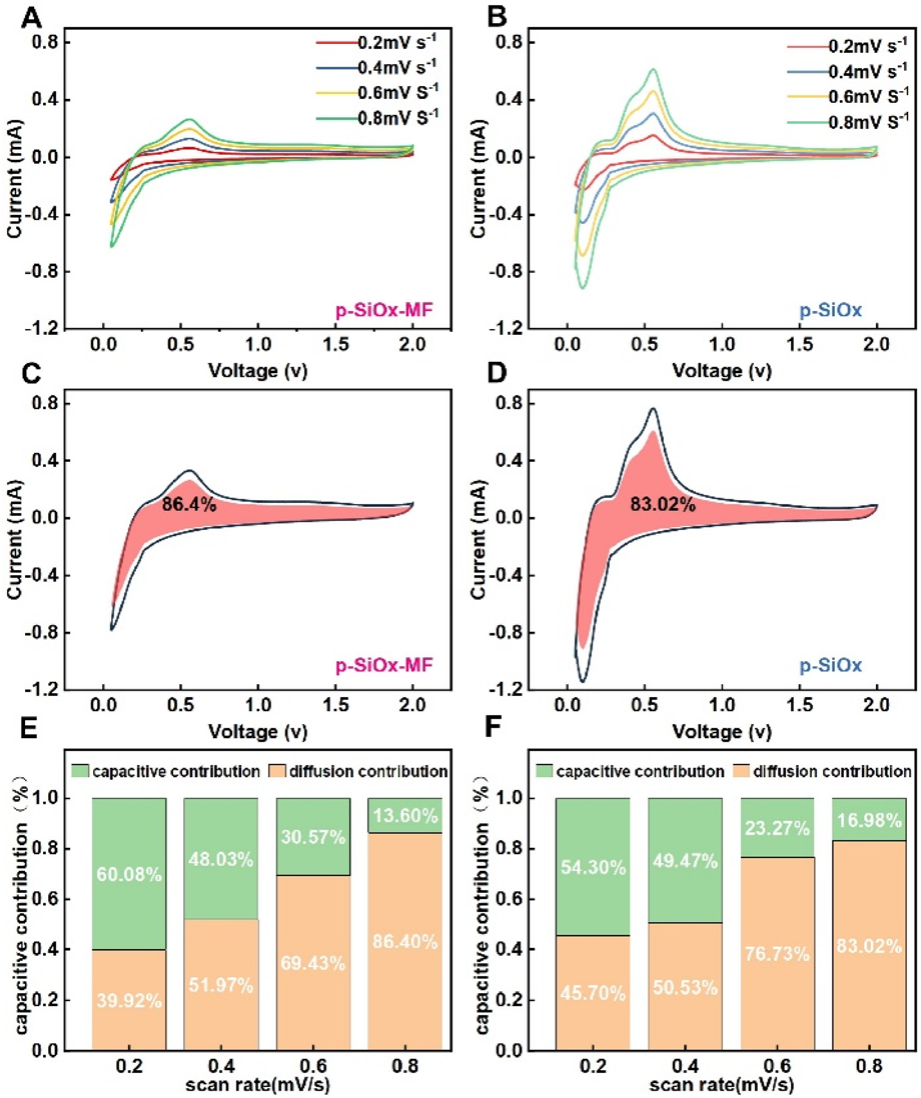
Capacity contribution by ions adsorption in the two materials. CV curves of (A) p-SiO_*x*_-MF and (B) p-SiO_*x*_ at different scan rates. Pseudocapacitive contribution at 0.8 mV s^−1^ of (C) p-SiO_*x*_-MF and (D) p-SiO_*x*_. Summary of pseudocapacitive contribution at various sweep rates of (E) p-SiO_*x*_-MF and (F) p-SiO_*x*_.

The CV profiles of p-SiO_*x*_ and p-SiO_*x*_-MF measured at various scan rates are displayed in Fig. 4A and B. The Li^+^ storage kinetic mechanism was analyzed using the classic power-law equation:^38^*i* = *aν*^*b*^log(*i*) = *b* log(*ν*) + log(*a*)where *a* is a pre-exponential constant and the *b*-value reflects the rate-determining kinetics. In general, *b* = 0.5 corresponds to pure diffusion-controlled lithium insertion, whereas *b* = 1.0 represents a surface pseudocapacitive-dominated process.

The fitted *b*-values are 0.91 for pristine p-SiO_*x*_ and 0.56 for p-SiO_*x*_-MF, respectively. The high *b*-value of pristine p-SiO_*x*_ reveals that its charge storage is dominated by surface pseudocapacitance owing to abundant exposed porous interfaces. By contrast, the lower *b*-value of p-SiO_*x*_-MF is close to 0.5, demonstrating that its lithiation/delithiation behavior is mainly governed by solid-state diffusion after introducing the rigid Mg_2_SiO_4_ inert skeleton.

To reveal the pseudo-capacities contribution of samples, the above equation can be converted as:^38^*i* = *k*_1_*ν* + *k*_2_*ν*^1/2^where *k*_1_*ν* and *k*_2_*ν*^1/2^ correspond to the pseudo-capacities-controlled contribution and diffusion-controlled contribution. As shown in Fig. 4C–F, the capacitive contributions of p-SiO_*x*_-MF and p-SiO_*x*_ are evolved into major contributions and achieve 86.4% and 83.2% at 0.8 mV s^−1^, respectively. The pseudo-capacities contribution values of both samples are almost the same and above 50%, indicating a high Li^+^ absorption efficiency.

Notably, although p-SiO_*x*_-MF shows a much lower *b*-value and intrinsically tends toward diffusion-dominated kinetics, it still delivers a slightly higher pseudocapacitive ratio than pristine p-SiO_*x*_ at high scan rates. This is attributed to the stable hierarchical porous architecture constructed by the Mg_2_SiO_4_ skeleton, which well maintains intact ion-transport channels and abundant accessible interfacial sites. Even under fast scanning conditions with limited diffusion time, the rigid framework enables efficient surface faradaic reactions and ion adsorption, thereby maintaining a high pseudocapacitive contribution and superior reaction kinetics.

**Fig. 5:**
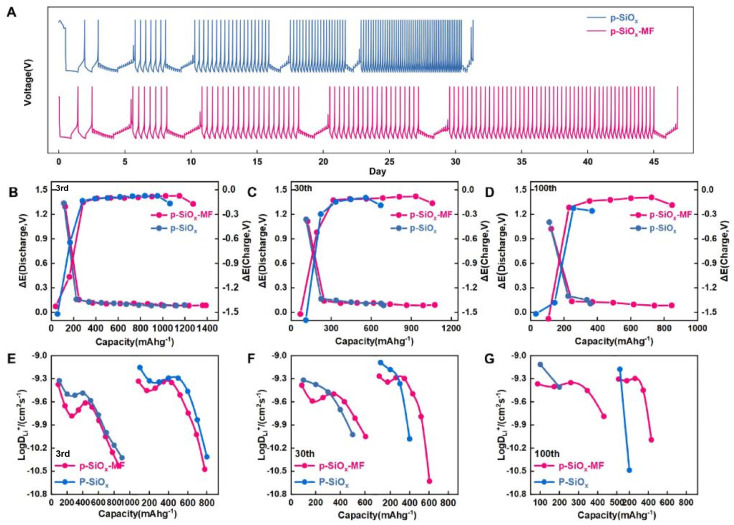
(A) Time–voltage curves of p-SiO_*x*_-MF and p-SiO_*x*_ during cycling (GITT tests performed at 3rd, 10th, 30th, 50th and 100th cycles). (B–D) Curves of polarization voltage (Δ*E*) *versus* specific capacity for circles and (E–G) comparison of Li^+^ diffusion coefficients between p-SiO_*x*_-MF and p-SiO_*x*_ at 3rd, 10th, 30th, 50th and 100th cycles.

The GITT was further employed to quantitatively evaluate the chemical diffusion coefficient of Li^+^ ions (*D*_Li^+^_, cm^2^ s^−1^). The calculation of Li^+^ diffusion coefficient in this work is performed under the semi-infinite linear diffusion assumption, which is the basic prerequisite for the classical GITT model and applicable to the long-term intermittent titration test of porous electrode materials. The overall GITT voltage profiles of p-SiO_*x*_ and p-SiO_*x*_-MF electrodes over 45 days of testing are shown in Fig. 5A. The two electrodes exhibit distinct long-term evolution behaviors: the p-SiO_*x*_ electrode shows gradually increasing voltage hysteresis and polarization over prolonged cycling, while the p-SiO_*x*_-MF electrode maintains stable voltage response throughout the test.

To further analyze the polarization evolution during cycling, the voltage hysteresis (Δ*E*) *vs.* capacity curves at the 3rd, 30th, and 100th cycles are presented in Fig. 5B–D. At the initial stage (3rd cycle), both electrodes show similar polarization characteristics. However, as cycling proceeds, the p-SiO_*x*_ electrode experiences a significant increase in voltage hysteresis, especially at the 100th cycle, indicating severe structural degradation and kinetic deterioration. In contrast, the p-SiO_*x*_-MF electrode retains its low-polarization behavior even after 100 cycles, demonstrating that the Mg_2_SiO_4_ framework effectively suppresses structural collapse and maintains stable electrochemical interfaces.

The *D*_Li^+^_ can be calculated based on the Fick's second law as follows.^39^
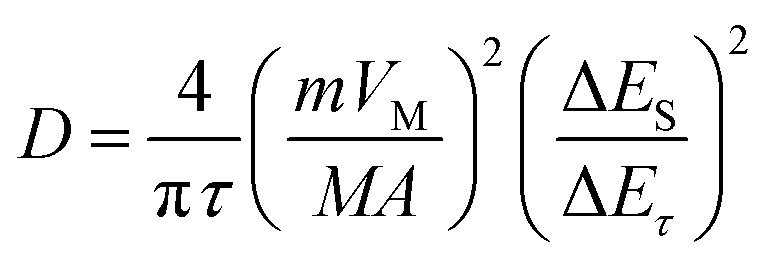
where *m* and *M* indicate the mass and molar mass of the electrode material, respectively. *V*_M_ (cm^3^ mol^−1^) refers to their molar volume, and *A* (cm^2^) stands for their active area. Δ*E*_S_, the change of steady state voltage; and Δ*E*_*τ*_, the total change of cell voltage during a current pulse, neglecting the ohmic drop. The evolution of Li^+^ diffusion coefficients of the anodes during cycling is presented in Fig. 5E–G. At the 3rd cycle, both electrodes exhibit comparable calculated *D*_Li^+^_ values, with an average *D*_Li^+^_ of approximately 3.42 × 10^−10^ cm^2^ s^−1^.

The evolution of Li^+^ diffusion coefficients at different cycles is plotted in Fig. 5E–G. At the 3rd cycle, both electrodes show comparable *D*_Li^+^_ values, indicating similar initial ion transport kinetics. With cycling progressing to the 30th and 100th cycles, a clear divergence emerges: the *D*_Li^+^_ of the p-SiO_*x*_ electrode drops sharply, which is attributed to the collapse of the porous structure and blockage of ion transport channels caused by repeated volume expansion. On the contrary, the p-SiO_*x*_-MF electrode maintains significantly higher and more stable *D*_Li^+^_ values throughout the 100 cycles. This directly confirms that the rigid Mg_2_SiO_4_ framework effectively preserves the interconnected porous pathways, ensuring persistent fast Li^+^ migration kinetics. Such stable porous structure configuration is the key to the improved cycling and kinetic performance of the composite anode.

As shown in Fig. S5, p-SiO_*x*_ exhibits a relatively low initial impedance (32.1 Ω), owing to its porous structure which provides shorter ion transport pathways, indicating a favorable initial electronic and ionic conductive network. In contrast, p-SiO_*x*_-MF demonstrates a higher initial impedance (98.49 Ω) due to the introduction of an inert framework, where the inherently low electrical conductivity of the framework partially blocks ion transport channels and increases charge transfer resistance. During cycling, p-SiO_*x*_ reaches an impedance of 85.24 Ω after 5 cycles, reflecting an increase of 165.5%, which primarily stems from volume expansion during charge/discharge processes, leading to particle cracking, poor contact, and repeated rupture-regrowth of the SEI layer that causes its continuous thickening. By comparison, p-SiO_*x*_-MF exhibits an impedance of 144.9 Ω after cycling with a relative increase of only 47.1%, showing significantly improved stability.

The inert Mg_2_SiO_4_ phase possesses relatively low intrinsic electronic conductivity, which inevitably leads to a slight increase in initial electrode impedance after introducing the rigid framework. Nevertheless, the interconnected Mg_2_SiO_4_ skeleton provides reliable mechanical support for SiO_*x*_/Si active particles, effectively inhibiting structural pulverization, avoiding the fracture of internal conductive network, and alleviating the excessive growth of SEI film during repeated lithiation/delithiation process. Although the Mg_2_SiO_4_ framework sacrifices partial initial electronic conductivity, it maintains stable ion/electron transport pathways in long-term cycling, which is crucial for improving the cycling durability and structural stability of porous SiO_*x*_/Si anode.^40,41^

The Si XPS characterization in Fig. S6 shows that the inert framework effectively stabilizes metallic silicon, preventing its complete oxidation, thereby enhancing the electrochemical performance and cycling stability of the material. The SEM image in Fig. S7 clearly demonstrates that p-SiO_*x*_-MF retains a certain porous structure due to the presence of the inert framework. Moreover, the HRTEM image of p-SiO_*x*_-MF after 5 cycles clearly displays lattice fringes corresponding to the Si(222) plane with a spacing of 0.19 nm, and those of Mg_2_SiO_4_(121) with a spacing of 0.23 nm (Fig. S8).

As an electrochemically inert phase, Mg_2_SiO_4_ itself contributes no Li storage capacity and introduces additional inactive mass to the electrode system. The introduction of such inert component inevitably endows the composite with a relatively high initial charge transfer impedance. This is directly reflected in its lower initial coulombic efficiency and inferior first-cycle specific capacity. Even so, the rigid Mg_2_SiO_4_ skeleton performs an irreplaceable mechanical supporting function, which effectively alleviates the enormous volume fluctuation of SiO_*x*_/Si active substances, inhibits irreversible pore collapse and particle pulverization, and maintains the integrity of the porous network during prolonged cycling. Although the introduction of Mg_2_SiO_4_ inevitably sacrifices part of the energy density, its unique rigid skeleton effect can effectively stabilize the porous structure of the SiO_*x*_/Si anode during prolonged Li insertion and extraction. Benefiting from this structural protection, the composite anode exhibits superior cycling stability compared with pristine porous SiO_*x*_ (Fig. S9). Such rigid framework support is therefore essential for maintaining and enhancing the long-term cycling performance of porous silicon-based anodes.

## 4 Conclusions

In summary, a hierarchical porous SiO_*x*_/Si composite anode embedded with inert Mg_2_SiO_4_ framework was successfully fabricated *via* a facile magnesiothermic reduction using natural diatomite. The *in situ* formed Mg_2_SiO_4_ rigid framework acts as a stable mechanical support, effectively mitigating volume expansion, preventing pore collapse, and preserving structural integrity during cycling. The optimized electrode delivers a high reversible capacity of 775 mAh g^−1^ after 100 cycles, superior to pristine porous SiO_*x*_, along with enhanced Li^+^ diffusion kinetics and suppressed SEI growth. This work establishes a low-cost, scalable route for robust porous Si-based anodes, clarifies the stabilization mechanism of silicate inert skeletons, and offers practical inspiration for high-performance lithium-ion battery anodes.

## Conflicts of interest

There are no conflicts to declare.

## Supplementary Material

RA-016-D6RA03303B-s001

## Data Availability

The data that support the findings of this study are available from the corresponding authors upon reasonable request. The data supporting this article have been included as part of the supplementary information (SI). Supplementary information: SI_1: XPS spectra of O1s; SI_2: XPS survey spectrum of the as-prepared p-SiO_*x*_-MF composite; SI_3: SEM images and linear sweep results of SiO_*x*_-MF and p-SiO_*x*_-MF; SI_4: EDS mapping of p-SiO_*x*_-MF; SI_5: Nyquist plots of p-SiO_*x*_-MF and p-SiO_*x*_ with equivalent circuit model; SI_6: XPS profiles of Si 2p of p-SiO_*x*_ and p-SiO_*x*_-MF after the 5th cycles (spectra measured in the middle of the film after etching and charge-corrected); SI_7: post-5th-cycles SEM images of p-SiO_*x*_-MF and SiO_*x*_; SI_8: post-5th-cycles TEM image of p-SiO_*x*_-MF; SI_9: impact of periodic GITT testing on long-cycle stability. See DOI: https://doi.org/10.1039/d6ra03303b.
