# Supplementary material for: Construction of an inert framework in porous SiOx/Si anodes for high-performance Li-ion batteries
Source: RSC Adv. 2026 Jun 2;16(33):29973–80. doi: 10.1039/d6ra03303b (PMC13231434; doi:10.1039/d6ra03303b)
Supplement: RA-016-D6RA03303B-s001 [file RA-016-D6RA03303B-s001.pdf]

Supplementary Information (SI) for RSC Advances.
This journal is © The Royal Society of Chemistry 2026

Supporting Information

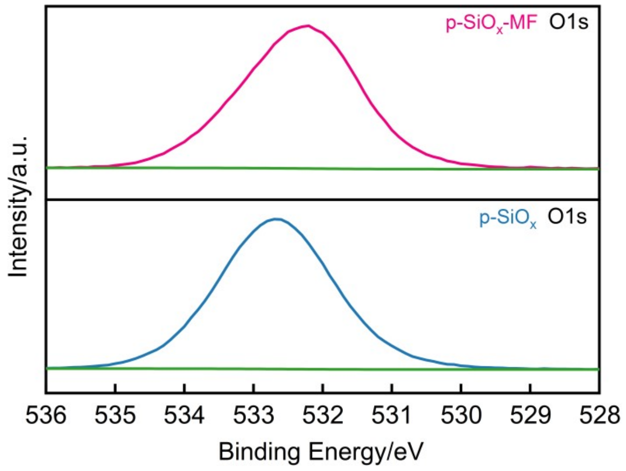

Figure S1. XPS spectra of O1s.

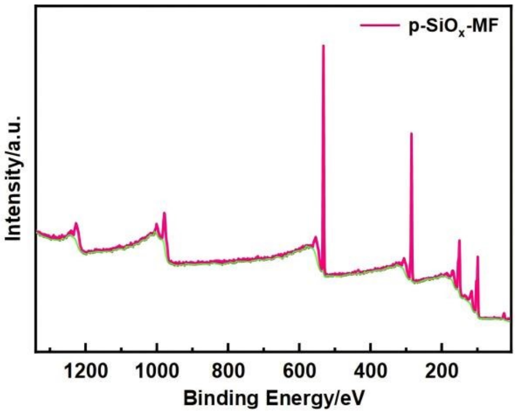

Figure S2. XPS survey spectrum of the as-prepared p-SiOx-MF composite.

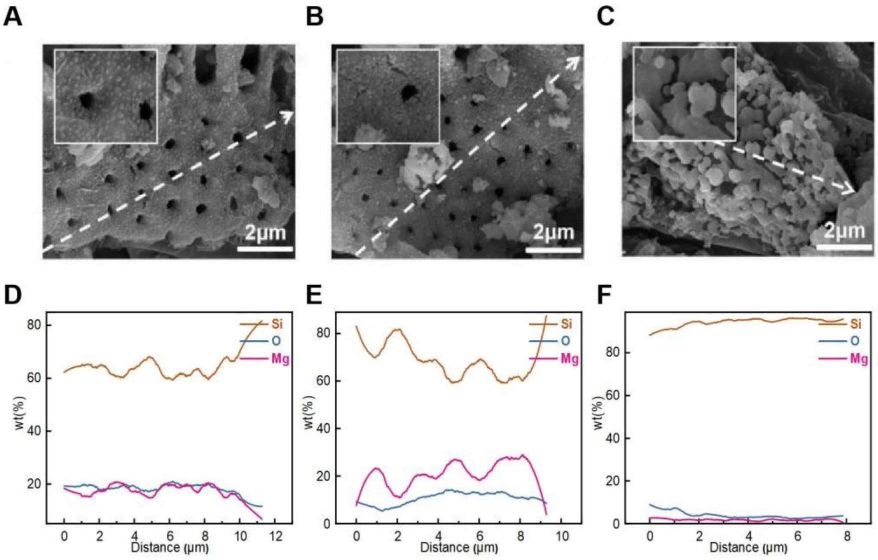

Figure S3. SEM image of (a) SiOx-MF and (b) p-SiOx-MF and (c) SiOx-MF. linear sweep of (d) SiOx-MF and (e) p-SiOx-MF and (f) SiOx-MF.

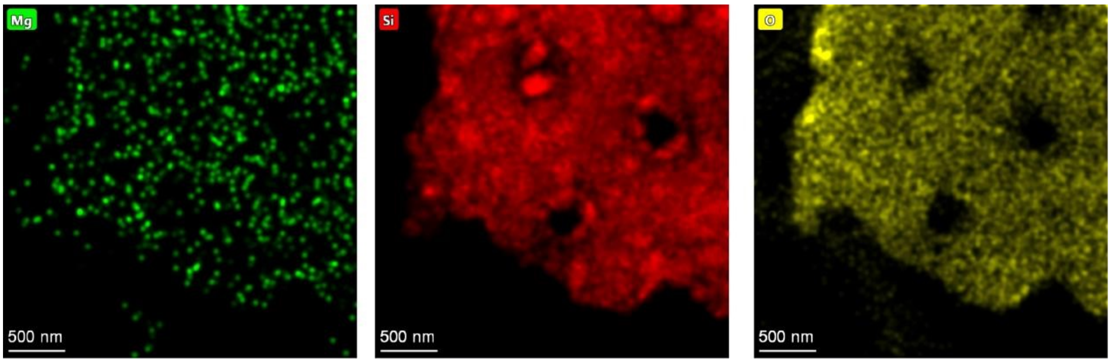

Figure S4. Corresponding EDS mapping of p-SiOx-MF.

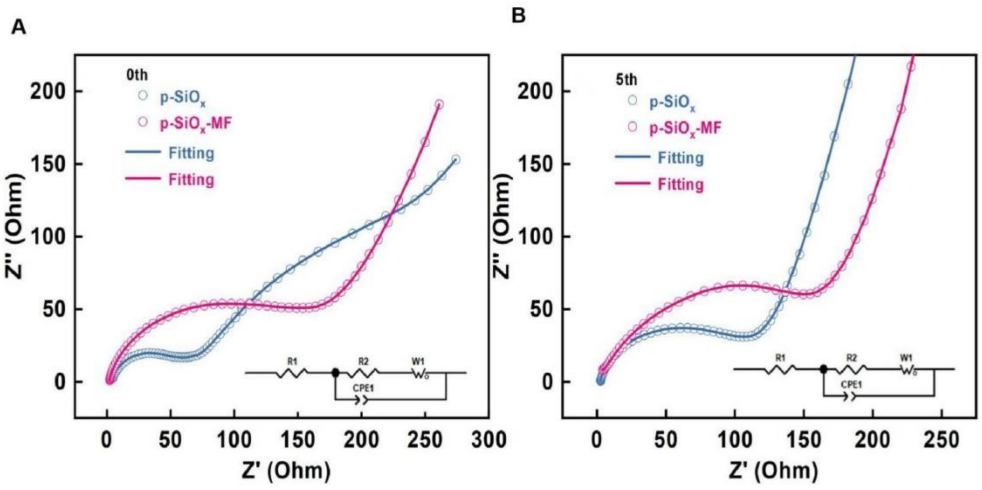

Figure S5. Nyquist plots of p-SiOx-MF and p-SiOx insert shows the equivalent circuit model of the studied system.

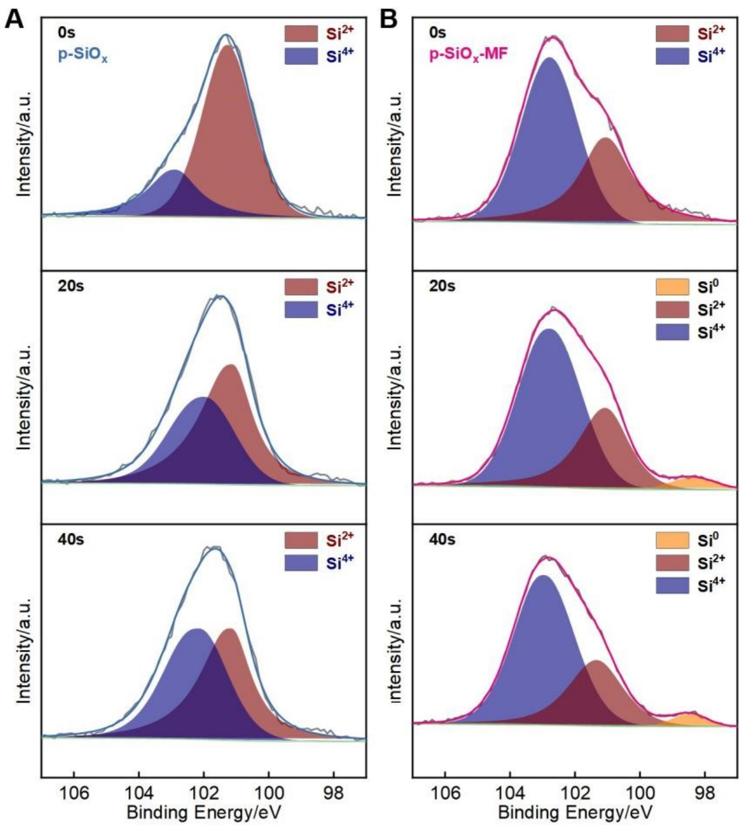

Figure S6. XPS profiles of Si 2p of (A) p-SiOx and (B)p-SiOx-MF after the 5th cycles. All spectra were measured in the middle of the film after etching and then charge-corrected.

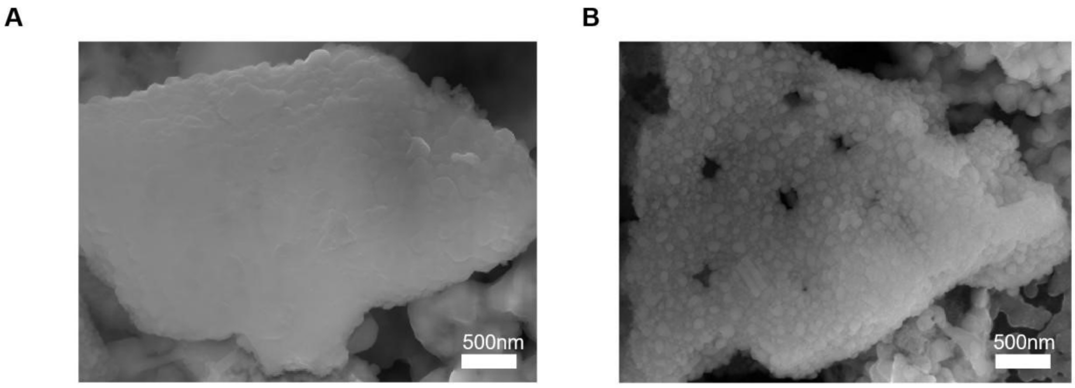

Figure S7. SEM image of (A)p-SiOx-MF and (B) SiOx after the 5th cycles.

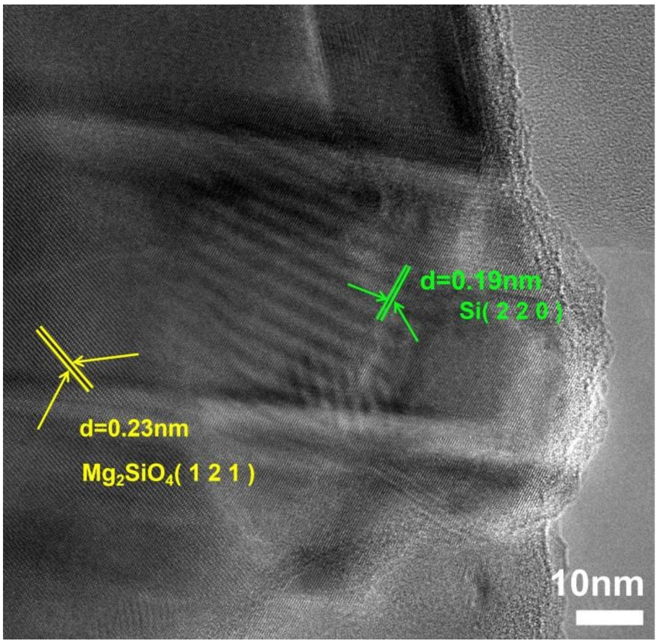

Figure S8. TEM image of p-SiOx-MF after the 5th cycles.

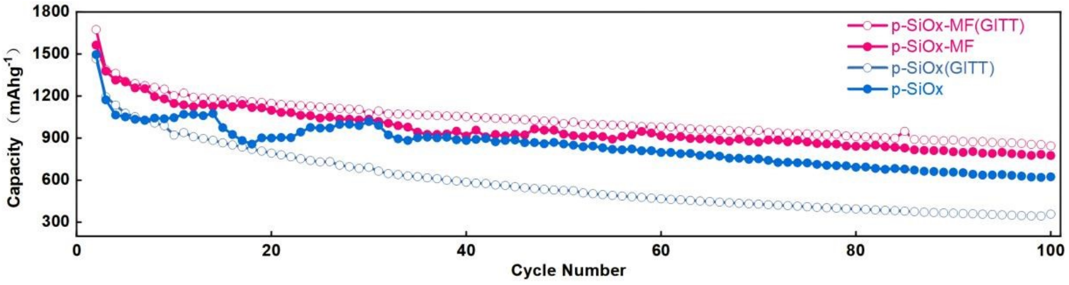

Figure S9. Impact of periodic GITT testing on long-cycle stability (3rd, 10th, 30th, 50th, and 100th tests constitute GITT evaluations).

TableS1. Comparison of different working performance

Material System	Initial Coulombic Efficiency (%)	Reversible Capacity (mAh/g)	Cycling Performance
**p-SiOx-MF（This work）**	**56**	**2494**	**775.8atmAhg^{-1} after 100 cycles**
Ji et al. (2024) d-SiO/C/LSO	90.3	1465	45.2% capacity retention after 100 cycles at 0.5C in half-cell; 86.5% retention after 100 cycles in NCM811 full-cell
Li et al. (2023) High-density Si-C composite	83.5–85.0	~600	96.2% retention over 200 cycles at 0.5C; 70.6% retention over 200 cycles in LFP full-cell
Liu et al. (2025) Si@h-SiO_x/C	~69.0	1520	70% retention over 300 cycles at 1 A/g; 94.4% retention for 300 cycles in pouch cell
Mamiya et al. (2023) SiO-C stacked film	—	2188.8	94% capacity retention over 500 cycles at 0.1C
Sun et al. (2023) SiO_x/C with different carbon microstructures	69–85	700–900	92.2%–98.5% retention after 100 cycles
Youn et al. (2024) Porous Si/Ca-Si-O	86.4	964.3	55% capacity retention over 200 cycles
Single carbon-coated SiO	76.7	1737	33.3% retention after 100 cycles at 0.5C
Single LiH-prelithiated SiO	81.0	1438	Only 1.2% retention after 100 cycles at 0.5C
Commercial graphite	92–94	372	≥90% retention after 100 cycles at

Material System	Initial Coulombic Efficiency (%)	Reversible Capacity (mAh/g)	Cycling Performance
			1C

References:

1 S. Ji, R. Song, H. Yuan, D. Lv, L. Yang, J. Luan, D. Wan, J. Liu and C. Zhong, Journal of Electroanalytical Chemistry, 2024, 959, 118141.

2 H. Li, Z. Chen, Z. Kang, W. Liu and Y. Chen, Energy Storage Materials, 2023, 56, 40–49.

3 Z. Liu, S. Wang, M. Zheng, Y. Zhang, R. Yu, X. Chen, W. Xiong, Z. Xu, Z. Zhuang, Y. Xia and L. Shen, Advanced Materials, 2026, 38, e17989.

4 M. Mamiya and J. Akimoto, Results in Chemistry, 2023, 5, 100815.

5 Q. Sun, J. Li, M. Yang, S. Wang, G. Zeng, H. Liu, J. Cheng, D. Li, Y. Wei, P. Si, Y. Tian and L. Ci, Small, 2023, 19, 2300759.

6 D. Youn, N. G. Kim, J. Y. Kim, S. T. Kim, D. J. Chung and H. Kim, Journal of Energy Storage, 2024, 89, 111641.

7 H. Zhen, F. Meng, J. Gao, Y. Liu and X. Liu, Small, 2023, 19, 2300500.

8 M. Li, D. Lu, J. Wang, S. Zhang, L. Lv, B. Ma, H. Zhu, L. Li, S. Yang, Z. Li, Y. Wu, J. Qi, L. Fan, R. Li, L. Chen, T. Deng and X. Fan, Nat Commun, 2026, 17, 3953.

9 Y. Li, J. Guo, K. Pedersen, L. Gurevich and D.-I. Stroe, Journal of Energy Chemistry, 2023, 80, 237–246.
